# Racial and ethnic differences in suicidal behavior and mental health service use among US adults, 2009–2020

**DOI:** 10.1017/S003329172200280X

**Published:** 2023-09

**Authors:** Tanner J. Bommersbach, Robert A. Rosenheck, Taeho Greg Rhee

**Affiliations:** 1 Department of Psychiatry and Psychology, Mayo Clinic, Rochester, MN, USA; 2 Department of Psychiatry, Yale School of Medicine, New Haven, CT, USA; 3 New England Mental Illness, Research Education, and Clinical Center, VA Connecticut Healthcare System, West Haven, CT, USA; 4 Department of Public Health Sciences, University of Connecticut School of Medicine, Farmington, CT, USA

**Keywords:** Ethnicity, mental health, race, suicidal ideation, suicide attempt, suicide

## Abstract

**Background:**

While suicide rates have recently declined for White individuals, rates among Black and Hispanic individuals have increased. Yet, little is known about racial/ethnic differences in precursors to suicide, including suicidal ideation (SI) and suicide attempts (SA).

**Methods:**

Data from 2009–2020 National Survey of Drug Use and Health (NSDUH) consisted of non-institutionalized US civilians aged ⩾18 (*n* = 426 008). We compared proportions of White, Black, and Hispanics among adults reporting no past-year suicidal thoughts/behavior, SI, and SA. Multivariable-adjusted analyses were used to evaluate the independence of observed racial/ethnic differences in past-year SI, SA, and mental health service use.

**Results:**

In the entire sample, 20 791 (4.9%) reported past-year SI only and 3661 (0.9%) reported a SA. Compared to White individuals, Black and Hispanic individuals were significantly less likely to report past-year SI [OR 0.73 (95% CI 0.69–0.77); OR 0.75 (95% CI 0.71–0.79), respectively], but more likely to report a past-year SA [OR 1.45 (95% CI 1.28–1.64); OR 1.19 (95% CI 1.04–1.37), respectively] even after multivariable adjustment. Black and Hispanic individuals were significantly less likely to use mental health services, but the lack of significant interactions between race/ethnicity and SI/SA in association with service use suggests differences in service use do not account for differences in SI or SA.

**Conclusions:**

Black and Hispanic individuals are significantly less likely than White individuals to report SI but more likely to report SAs, suggesting differences in suicidal behavior across race/ethnicity that may be impacted by socio-culturally acceptable expressions of distress and structural racism in the healthcare system.

## Introduction

For the first time in two decades, the suicide rate in the US declined for consecutive years from 2018 to 2020 (Garnett, Curtin, & Stone, 2022). Recent data, however, have revealed potentially concerning differences in suicide rates among racial and ethnic groups. The decline in suicide appears primarily driven by a decline among non-Hispanic White individuals, while rates among Black and Hispanic individuals have continued to rise (Curtin, 2022; Garnett et al., 2022). Although suicide is considered an outcome most impacting White individuals, especially White males, suicide rates among Black individuals increased by 30% from 2014 to 2019 (Ramchand, Gordon, & Pearson, 2021).

Two well-documented risk factors for suicide are suicidal ideation (SI) and prior suicide attempts (SA) (Bostwick, Pabbati, Geske, & McKean, 2016; Harris & Barraclough, 1997). Existing literature suggests that suicidality may develop along a continuum, from less severe forms, including thoughts of death or SI, to more severe behavior, including SAs (Sveticic & De Leo, 2012). In 2005, the National Comorbidity Survey (NCS) reported that 34% of individuals with SI progressed to developing a plan and 72% progressed from a plan to making an attempt (Kessler, Borges, & Walters, 1999; Sveticic & De Leo, 2012). Intervening at earlier points along this continuum may thus prevent progression to more severe forms of behavior.

Little is known, however, about the differential prevalence of SI and SAs among under-represented racial and ethnic groups as compared to White individuals and whether there are differences in the percentage of individuals in these groups who receive mental health care or reasons some don't. One potential explanation for why suicide among Black and Hispanic individuals is increasing is that these groups may be less likely to receive mental health treatment at earlier points along the suicide continuum (Han, Compton, Gfroerer, & McKeon, 2014; Congressional Black Caucus, 2019). To better understand why suicide may be increasing among these groups and to develop effective preventive interventions along the suicide continuum, it is important not only to examine differential rates of SI and SAs among these groups but also to identify potential disparities in receipt of mental health services among racial and ethnic subgroups experiencing SI and SAs.

Since the US does not have a population-based surveillance system for suicidal thoughts or attempts, the field relies on estimates from epidemiologic surveys, which have presented mixed and outdated results. The Epidemiologic Catchment Area (ECA) study of 1980–1984 found that White individuals were 1.7 times more likely than Black and Hispanic individuals to report SAs, while the NCS of 1990–1992 and NCS-R of 2001–2003 found no significant difference in past-year SI or SAs between racial/ethnic subgroups (Kessler, Berglund, Borges, Nock, & Wang, 2005; Moscicki et al., 1988). In addition, the 2001–2003 National Survey of American Life (NSAL) provides prevalence estimates of SI and SAs for African Americans and Caribbean Blacks and the 2002–2003 National Latino and Asian American Survey (NLAAS) provides prevalence estimates for Asian Americans and Latinos, but it is difficult to directly compare estimates across surveys (Cheng et al., 2010; Joe, Baser, Breeden, Neighbors, & Jackson, 2006; Joe, Baser, Neighbors, Caldwell, & Jackson, 2009). Thus, in the context of rising suicide rates among Black and Hispanic individuals, research is needed on current racial and ethnic differences in SI, SAs, and receipt of mental health treatment.

The current study uses nationally-representative data from the 2009–2020 National Survey on Drug Use and Health (NSDUH) (Substance Abuse and Mental Health Services Administration, 2020b) to compare proportions of non-Hispanic White, non-Hispanic Black, and Hispanic adults among those with no suicidal behavior, past-year SI, and past-year SAs and whether these differences are accounted for by co-morbidities. We also examine differences in mental health service use among racial/ethnic groups who report recent SI or SA and reasons for not using services. Through these analyses, we hope to shed light on rising rates of suicide among Black and Hispanic individuals and inform upstream interventions along the suicide continuum.

## Methods

### Data source and study sample

We used 2009–2020 data from NSDUH, which collects systematic information about drug use, health, and health services among non-institutionalized US civilians aged ⩾12 (Substance Abuse and Mental Health Services Administration, 2020b). NSDUH employs a multi-stage stratified sampling design to produce nationally-representative estimates. We limited our sample to adults aged ⩾18 who self-identified as non-Hispanic Black (hereafter referred to as Black), Hispanic, or non-Hispanic White (referred to as White) because suicide-related questions were only asked among adults (*n* = 426 008 unweighted). Participants received $30.00 for completion of the survey. The annual mean weighted response rate in 2009–2020 ranged from 60.4% to 75.6%. Response rates for racial and ethnic groups varied depending on the survey year but were comparable across years. For example, in the 2018 NSDUH, the weighted response rates for Hispanic, non-Hispanic Black, and non-Hispanic White individuals were 67, 73.1, and 66.2%, respectively, and 64.4, 70.6, and 64.7%, respectively, in the 2019 NSDUH (Substance Abuse and Mental Health Services Administration, 2020a).

This study was exempted from review by the Institutional Review Board at Yale School of Medicine as data are publicly available, de-identified. Study procedures followed the Strengthening the Reporting of Observational Studies in Epidemiology (STROBE) guideline and updated guidance for reporting of race/ethnicity in medical research (Flanagin, Frey, Christiansen, & Committee, 2021). Further details of the survey are available on the NSDUH website (Substance Abuse and Mental Health Services Administration, 2020b).

### Measures

*Suicidal thoughts and behaviors* were assessed by several questions. SI was assessed by asking whether respondents had seriously thought about killing themselves (‘At any time in the past 12 months, did you seriously think about trying to kill yourself?’) or made a plan to kill themselves (‘During the past 12 months, did you make any plans to kill yourself?’). Those who responded yes to either question were asked if they had attempted to kill themselves in the past 12 months (SAs) (‘During the past 12 months, did you try to kill yourself?’) (Bommersbach, Rosenheck, & Rhee, 2022c; Substance Abuse and Mental Health Services Administration, 2018).

*Sociodemographic characteristics* included age, sex, marital status, education, employment, annual family income, health insurance coverage, and urban residence (based on metropolitan statistical areas) (US Census Bureau, 2020).

*Past-year psychiatric disorders:* Major depressive episode (MDE) was measured by a series of questions based on the *Diagnostic and Statistical Manual of Mental Disorders, Fourth Edition* (DSM-IV) criteria for MDE (American Psychiatric Association, 1994). Substance use disorders (SUDs) were derived from questions addressing formal diagnostic criteria for substance abuse and dependence as defined in DSM-IV (American Psychiatric Association, 1994), which included alcohol, tobacco, cannabis, cocaine/crack, heroin/pain reliever, hallucinogen, stimulant, sedative, and an aggregate indicator of any substance abuse or dependence (excluding alcohol and tobacco).

*Past-year mental health service use* was assessed by questions concerning receipt of any outpatient or inpatient mental health services or prescription medications for mental health problems. Among respondents who did not report receipt of any mental health services, data were gathered about the subjective reasons for not receiving services from a list of 11 potential options.

### Data analysis

First, the sample was divided into three groups: individuals with no past-year suicidal thoughts/behavior, past-year SI only, or a past-year SA. Next, bivariate logistic regression was used to compare the sociodemographic and clinical characteristics of the three groups, including whether the racial/ethnic subgroups were over or under-represented in the three groups. Odds ratios (OR) and 95% confidence intervals (CI) were reported.

Next, multivariable-adjusted analyses were conducted to determine if significant differences between the three comparison groups in the bivariate analyses remained significant after controlling for other factors associated with past-year suicidal thoughts/behaviors. In these models, past-year SI and SA were dependent variables while the primary independent variable of interest was racial/ethnic subgroups, and covariates included sociodemographic and clinical factors that had emerged as significantly associated with suicidal thoughts/behaviors in the bivariate analysis as well as survey year to identify period effects.

Next, we examined past-year mental health service use by racial/ethnic subgroup and year separately for those reporting SI and SAs. We further examined service use in a multivariable-adjusted interaction analysis in which mental health service use was the dependent variable. Independent variables included (1) main effects for Black and Hispanic individuals, (2) dichotomous variables representing SI and SA, and four interaction terms of each race by each type of suicidal thoughts/behavior. These analyses sought to determine whether individuals who are Black or Hispanic had greater or lesser likelihood of receiving mental health services, whether SI or SA were associated with service use, and whether the relationship of SI and SA to service use differed by racial/ethnic group.

Finally, among respondents who reported past-year SI or a SA and did not report receiving any mental health services, we compared the racial/ethnic groups on reasons for not receiving services.

We used Stata version 16.1 MP/4-Core for all analyses. We accounted for NSDUH complex survey design using *svy* commands to account for multi-stage, complex survey sampling techniques used in the data collection. We set *p* value <0.05 as the test of statistical significance.

## Results

### Characteristics of the study sample

In the entire NSDUH sample of adults aged ⩾18 from 2009 to 2020 (*n* = 426 008 unweighted, representing over 220 million adults nationwide), 20 791 (4.9%; representing 8 007 155 individuals) reported past-year SI alone, 3661 (0.9%; representing 1 123 676 individuals) reported past-year SI and SAs, and 401 556 individuals reported no suicidal thoughts/behavior. From 2009 to 2020, there was a significant increase in past-year SI, from 14.8% to 20.5% of all respondents (OR 1.33, 95% CI 1.21–1.45) but no significant change in the proportion of individuals with SI who reported past-year SAs (OR 1.14, 95% CI 0.92–1.40). We did not find any significant change in past-year SI or SAs between years 2019 and 2020 (i.e. pre- *v.* post-Covid-19 pandemic) for the study sample.

### Bivariate comparison by suicidal thoughts/behavior

Compared to White individuals, Black individuals were significantly less likely to report past-year SI but among individuals with past-year SI, were more likely to report a past-year SA, as compared to no suicidal thoughts/behavior (Table 1, columns 4 and 5). Similarly, Black individuals with past-year SI were much more likely to report a past-year SA than past-year SI alone (OR 1.99, 95% CI 1.74–2.26) (Table 1, column 6). Hispanic individuals demonstrated similar patterns as they were significantly less likely to report past-year SI but were more likely to report a past-year SA, as compared to White individuals.

**Table 1.** Socio-demographic and clinical characteristics by suicidal thoughts/behavior among 426 008 US adults, 2009–2020

	No suicidal behavior (1)	Suicidal ideation (2)	Suicide attempt (3)	Bivariate odds ratio (95% confidence interval)		
				(2) *v.* (1)	(3) *v.* (1)	(3) *v.* (2)
Sample size						
Unweighted	401 556	20 791	3661			
Weighted	211 282 271	8 007 155	1 123 676			
Race/ethnicity						
Non-Hispanic white	70.5%	76.4%	64.7%	Reference	Reference	Reference
Non-Hispanic black	12.8%	10.1%	17.0%	0.73 (0.69–0.77)***	1.45 (1.28–1.64)***	1.99 (1.74–2.26)***
Hispanic (white or black)	16.7%	13.5%	18.3%	0.75 (0.71–0.79)***	1.19 (1.04–1.37)*	1.59 (1.37–1.85)***
Age, years						
18–25	13.4%	27.3%	40.2%	2.07 (1.97–2.18)***	3.35 (2.93–3.84)***	1.62 (1.41–1.86)***
26–34	15.4%	19.4%	17.3%	1.28 (1.20–1.36)***	1.25 (1.05–1.50)*	1.25 (1.05–1.50)*
35–49	25.3%	24.9%	22.6%	Reference	Reference	Reference
50–64	25.9%	19.9%	13.9%	0.78 (0.72–0.85)***	0.60 (0.48–0.75)***	0.60 (0.48–0.75)***
⩾65	20.1%	8.5%	5.9%	0.43 (0.38–0.49)***	0.33 (0.24–0.45)***	0.33 (0.24–0.45)***
Sex						
Female	51.6%	53.2%	58.1%	Reference	Reference	Reference
Male	48.4%	46.8%	42.0%	0.94 (0.89–0.99)*	0.77 (0.69–0.86)***	0.82 (0.73–0.93)**
Marital status						
Married	52.8%	33.5%	21.4%	Reference	Reference	Reference
Widowed	6.2%	3.4%	3.7%	0.86 (0.71–1.03)	1.49 (1.01–2.20)*	1.73 (1.14–2.64)*
Divorced/separated	14.1%	17.6%	19.9%	1.96 (1.82–2.12)***	3.48 (2.92–4.15)***	1.77 (1.47–2.14)***
Never married	26.9%	45.5%	55.0%	2.67 (2.53–2.82)***	5.06 (4.47–5.74)***	1.90 (1.67–2.15)***
Education						
<High school	13.6%	12.7%	22.8%	Reference	Reference	Reference
High school or equivalent	28.2%	27.3%	34.0%	1.04 (0.95–1.13)	0.72 (0.62–0.83)***	0.69 (0.59–0.81)***
Some college	28.6%	35.9%	31.3%	1.34 (1.23–1.46)***	0.65 (0.56–0.75)***	0.48 (0.41–0.57)***
⩾Bachelor's degree	29.6%	24.1%	11.8%	0.87 (0.80–0.95)**	0.24 (0.19–0.29)***	0.27 (0.22–0.34)***
Employment						
Full-time	49.9%	42.4%	32.3%	Reference	Reference	Reference
Part-time	13.4%	17.1%	15.4%	1.51 (1.40–1.62)***	1.78 (1.53–2.07)***	1.18 (1.00–1.40)
Unemployed	4.8%	8.6%	14.2%	2.09 (1.91–2.27)***	4.54 (3.93–5.24)***	2.17 (1.85–2.56)***
Other	32.0%	32.0%	38.1%	1.18 (1.11–1.25)***	1.84 (1.61–2.10)***	1.56 (1.36–1.80)***
Family income						
<$20 000	16.9%	25.1%	35.7%	Reference	Reference	Reference
$20 000–$49 999	31.0%	33.3%	35.6%	0.72 (0.68–0.77)***	0.54 (0.48–0.61)***	0.75 (0.66–0.86)***
$50 000–$74 999	16.6%	14.9%	11.3%	0.60 (0.56–0.65)***	0.32 (0.27–0.38)***	0.53 (0.45–0.63)***
⩾$75 000	35.5%	26.8%	17.5%	0.51 (0.48–0.54)***	0.23 (0.20–0.27)***	0.46 (0.39–0.54)***
Insurance coverage						
Private plan	51.4%	47.0%	33.3%	Reference	Reference	Reference
Medicaid	8.0%	13.5%	23.4%	1.84 (1.72–1.98)***	4.50 (3.97–5.10)***	2.44 (2.13–2.79)***
Medicare	22.3%	15.2%	13.8%	0.75 (0.69–0.81)***	0.96 (0.79–1.16)	1.28 (1.04–1.57)*
Other	3.8%	5.6%	5.5%	1.61 (1.45–1.78)***	2.23 (1.77–2.79)***	1.38 (1.09–1.76)**
Uninsured	14.5%	18.7%	24.0%	1.41 (1.34–1.49)***	2.56 (2.25–2.92)***	1.81 (1.58–2.08)***
Urban residence						
Non-metropolitan	15.7%	15.8%	17.9%	Reference	Reference	Reference
Small metropolitan	30.7%	33.0%	33.6%	1.07 (1.00–1.13)*	0.96 (0.82–1.13)	0.90 (0.77–1.05)
Large metropolitan	53.7%	51.2%	48.5%	0.95 (0.89–1.01)	0.79 (0.68–0.92)**	0.84 (0.71–0.99)*
Past-year major depressive episode	5.3%	50.0%	52.6%	17.92 (17.06–118.82)***	19.85 (17.70–22.27)***	1.11 (0.98–1.25)
Past-year substance use disorder						
Alcohol	6.1%	18.3%	25.2%	3.47 (3.26–3.70)***	5.21 (4.67–5.81)***	1.50 (1.32–1.71)***
Tobacco	8.2%	17.2%	24.4%	2.33 (2.19–2.48)***	3.62 (3.24–4.05)***	1.56 (1.37–1.76)***
Cannabis	1.3%	6.2%	10.0%	5.04 (4.66–5.45)***	8.54 (7.34–9.93)***	1.69 (1.44–2.00)***
Cocaine	0.3%	2.0%	4.4%	6.72 (5.53–8.18)***	15.05 (11.60–19.51)***	2.24 (1.63–3.08)***
Opioid	0.7%	4.3%	8.1%	6.84 (6.08–7.70)***	13.42 (11.23–16.03)***	1.96 (1.64–2.34)***
Hallucinogen	0.1%	0.5%	2.4%	6.31 (4.81–8.28)***	33.41 (24–12–46.28)***	5.30 (3.76–7.46)***
Stimulant	0.1%	1.0%	2.5%	7.34 (5.83–9.25)***	18.39 (13.54–24.99)***	2.51 (1.74–3.61)***
Sedative	0.2%	1.8%	4.3%	11.12 (9.24–13.37)***	26.88 (21.25–33.99)***	2.42 (1.81–3.24)***
Any SUD, excluding alcohol and tobacco	2.3%	12.3%	21.3%	5.97 (5.55–6.41)***	11.53 (10.28–12.94)***	1.93 (1.71–2.18)***
Survey year						
2009–2010	16.2%	14.8%	14.1%	Reference	Reference	Reference
2011–2012	16.4%	14.7%	15.9%	0.98 (0.89–1.08)	1.111 (0.92–1.35)	1.14 (0.92–1.41)
2013–2014	16.7%	15.9%	16.6%	1.04 (0.95–1.14)	1.14 (0.92–1.41)	1.10 (0.88–1.37)
2015–2016	16.9%	16.3%	18.2%	1.05 (0.96–1.15)	1.24 (1.03–1.50)*	1.18 (0.96–1.44)
2017–2018	17.1%	17.8%	18.6%	1.14 (1.04–1.25)**	1.26 (1.04–1.51)*	1.11 (0.91–1.35)
2019–2020	16.9%	20.5%	16.7%	1.33 (1.21–1.45)***	1.14 (0.92–1.40)	0.86 (0.69–1.07)

*Note*: Data are from National Survey on Drug Use and Health.

*<0.05; **<0.01; ***<0.001.

### Multivariable-adjusted comparison by suicidal thoughts/behavior

In the multivariable analysis adjusted for factors significantly associated with suicidal thoughts/behavior in the bivariate, Black and Hispanic individuals remained significantly less likely than White individuals to report past-year SI, as compared to no suicidal thoughts/behavior [adjusted OR (aOR) 0.71, 95% CI 0.66–0.76; aOR 0.70, 95% CI 0.66–0.75, respectively] (Table 2). Black individuals with past-year SI also remained significantly more likely to report a past-year SA as compared to no suicidal thoughts/behavior, and much more likely when compared to SI alone (aOR 1.65, 95% CI 1.41–1.94). However, while Hispanic individuals were no longer more likely than White individuals to report a past-year SA as compared to no suicidal thoughts/behavior, they remained significantly more likely to report a past-year SA, as compared to those with SI alone (aOR 1.36, 95% CI 1.17–1.57).

**Table 2.** Multivariable-adjusted logistic regression models examining factors associated with suicidal thoughts/behaviors among 426 008 US adults, 2009–2020

Reference group in a parenthesis.	Suicidal ideation *v.* no suicidal behavior	Suicide attempt *v.* no suicidal behavior	Suicide attempt *v.* suicidal ideation
Race/ethnicity (non-Hispanic white)			
Non-Hispanic black	0.71 (0.66–0.76)***	1.23 (1.06–1.42)**	1.65 (1.41–1.94)***
Hispanic, black or white	0.70 (0.66–0.75)***	0.99 (0.86–1.15)	1.36 (1.17–1.57)***
Age, years (35–49)			
18–25	1.54 (1.43–1.66)***	2.52 (2.12–3.00)***	1.66 (1.36–2.04)***
26–34	1.12 (1.04–1.21)**	1.15 (0.95–1.40)	1.07 (0.86–1.32)
50–64	0.81 (0.74–0.88)***	0.60 (0.47–0.76)***	0.79 (0.62–1.01)
⩾65	0.46 (0.39–0.54)***	0.30 (0.19–0.48)***	0.71 (0.45–1.10)
Sex (female)			
Male	1.14 (1.08–1.21)***	0.92 (0.81–1.04)	0.81 (0.71–0.92)**
Marital status (married)			
Widowed	0.93 (0.76–1.14)	1.40 (0.93–2.12)	1.45 (0.95–2.23)
Divorced/separated	1.29 (1.18–1.40)***	1.79 (1.48–2.16)***	1.43 (1.18–1.73)***
Never married	1.31 (1.22–1.42)***	1.39 (1.18–1.65)***	1.07 (0.88–1.28)
Education (<high school)			
High school or equivalent	1.01 (0.92–1.10)	0.82 (0.70–0.96)*	0.80 (0.67–0.95)*
Some college	1.15 (1.05–1.26)**	0.69 (0.59–0.81)***	0.61 (0.51–0.73)***
⩾Bachelor's degree	1.03 (0.93–1.14)	0.50 (0.40–0.62)***	0.48 (0.38–0.62)***
Employment (full-time)			
Part-time	1.14 (1.05–1.24)**	1.06 (0.89–1.25)	0.93 (0.78–1.12)
Unemployed	1.34 (1.21–1.47)***	1.77 (1.49–2.11)***	1.44 (1.20–1.73)***
Other	1.17 (1.10–1.25)***	1.47 (1.24–1.73)***	1.25 (1.06–1.47)**
Family income (<$20 000)			
$20 000–$49 999	0.97 (0.90–1.04)	0.95 (0.84–1.08)	1.02 (0.88–1.17)
$50 000–$74 999	0.87 (0.80–0.95)**	0.77 (0.63–0.93)**	0.87 (0.71–1.05)
⩾$75 000	0.84 (0.77–0.91)***	0.78 (0.66–0.92)**	0.89 (0.75–1.05)
Insurance coverage (private plan)			
Medicaid	1.10 (1.01–1.20)*	1.54 (1.31–1.81)***	1.44 (1.25–1.67)***
Medicare	1.43 (1.25–1.64)***	1.88 (1.39–2.53)***	1.31 (0.97–1.76)
Other	1.29 (1.14–1.46)***	1.51 (1.18–1.94)**	1.17 (0.91–1.52)
Uninsured	1.09 (1.01–1.16)*	1.33 (1.15–1.54)***	1.19 (1.03–1.37)*
Urban residence (non-metropolitan)			
Small metropolitan	1.04 (0.97–1.12)	0.94 (0.80–1.11)	0.92 (0.78–1.09)
Large metropolitan	1.00 (0.92–1.08)	0.84 (0.71–0.98)*	0.86 (0.72–1.03)
Past-year major depressive episode	13.54 (12.78–14.34)***	12.31 (10.86–13.95)***	1.05 (0.92–1.19)
Past-year substance use disorder			
Alcohol	1.79 (1.65–1.94)***	2.23 (1.96–2.54)***	1.37 (1.20–1.56)***
Tobacco	1.23 (1.14–1.34)***	1.43 (1.24–1.64)***	1.22 (1.06–1.42)**
Cannabis	1.60 (1.44–1.78)***	1.57 (1.29–1.90)***	1.03 (0.86–1.24)
Cocaine	1.54 (1.19–2.00)**	1.75 (1.28–2.39)***	1.13 (0.79–1.62)
Opioid	1.89 (1.57–2.27)***	2.01 (1.52–2.65)***	1.23 (0.97–1.55)
Hallucinogen	0.94 (0.64–1.38)	2.87 (1.72–4.78)***	2.60 (1.75–3.87)***
Stimulant	0.90 (0.64–1.27)	1.10 (0.72–1.70)	1.44 (0.97–2.13)
Sedative	1.61 (1.20–2.15)**	2.06 (1.39–3.05)***	1.37 (0.96–1.96)
Survey year (2009–2010)			
2011–2012	0.98 (0.88–1.09)	1.14 (0.92–1.41)	1.19 (0.94–1.50)
2013–2014	1.08 (0.98–1.20)	1.25 (0.99–1.57)	1.17 (0.92–1.48)
2015–2016	1.11 (1.01–1.22)*	1.39 (1.13–1.70)**	1.28 (1.03–1.59)*
2017–2018	1.18 (1.07–1.30)**	1.42 (1.15–1.74)**	1.18 (0.96–1.46)
2019–2020	1.31 (1.19–1.44)***	1.26 (1.01–1.58)*	0.97 (0.77–1.23)

*Note*: Data are from National Survey on Drug Use and Health.

*<0.05; **<0.01; ***<0.001.

In subsequent interaction analyses, there was no significant interaction of race/ethnicity by survey year, by sex, or by either past-year SI or SA. Thus, the effects of race did not differ over the years, across sexes, or types of suicidal behavior. There was no consistent time trend that differentiated suicidality among Black or Hispanic individuals from Whites.

### Mental health service use

**Fig. 1.**
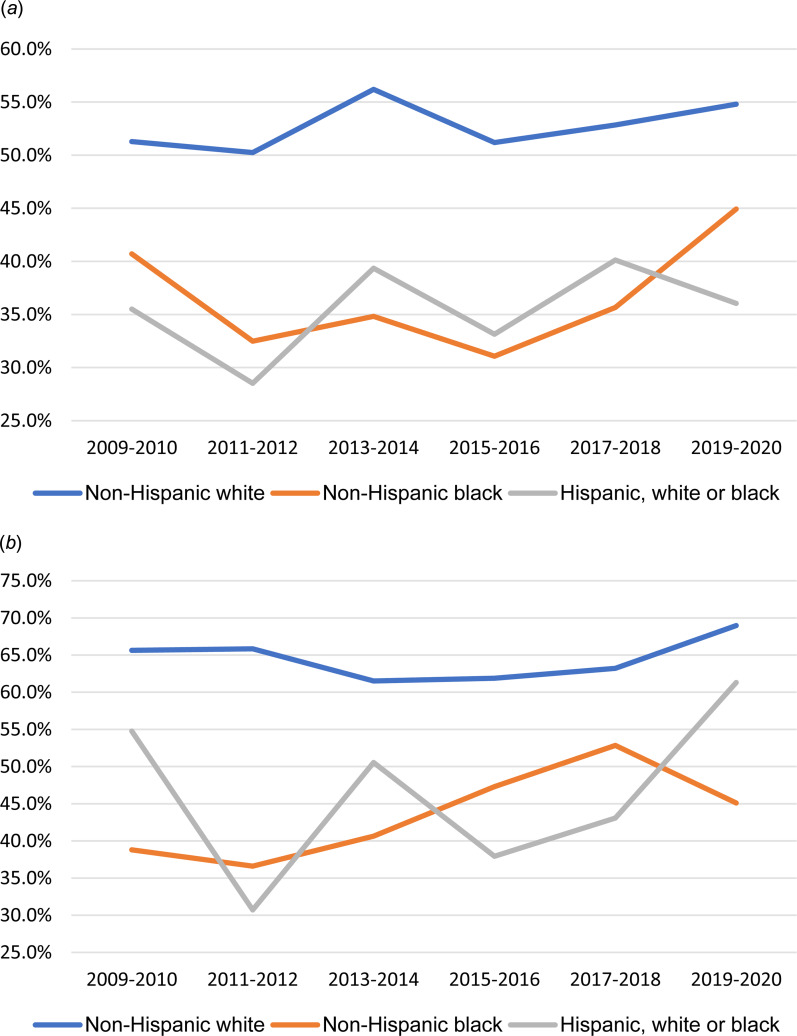
Trends of receiving any mental health care in the past year by suicidal behaviors and race/ethnicity, 2009–2020. (*a*) Among US adults with past-year suicidal ideation, 2009–2020. (*b*) Among US adults with past-year suicide attempt, 2009–2020. *Note*: Data are from National Survey on Drug Use and Health.

Among individuals who reported past-year SI or SA from 2009 to 2020, lower percentages of Black and Hispanic individuals reported receiving mental health services, compared to White individuals (Fig. 1*a* and *b*).

Multivariable-adjusted interaction analysis of past-year mental health service use (Table 3) showed significant main effects for suicidal thoughts/behavior on past-year mental health service use as well as main effects for race and ethnicity with Black and Hispanic individuals significantly less likely than White individuals to report receiving any mental health services (aOR 0.39, 95% CI 0.37–0.41; aOR 0.45, 95% CI 0.43–0.47, respectively). However, the interactions of race/ethnicity and past-year SI or SA in association with mental health service use were *not* significant, indicating that the significant relationship of SI and SA to mental health service use did not differ by racial/ethnic group. The results can also be understood as failing to support the possibility that the lower levels of service use among Black and Hispanic individuals account for either their lower risk for reporting SI or greater risk for reporting a SA.

**Table 3.** Multivariable-adjusted interaction analyses of mental health service use and race/ethnicity on suicidal behaviors among 426 008 US adults, 2009–2020

Reference group in a parenthesis.	AOR (95% CI; *p*)
Suicidal behavior (none)	
Suicidal ideation (1)	2.45 (2.30–2.62; <0.001)
Suicide attempt (2)	3.69 (3.15–4.32; <0.001)
Race/ethnicity (non-Hispanic white)	
Non-Hispanic black (3)	0.39 (0.37–0.41; <0.001)
Hispanic, black or white (4)	0.45 (0.43–0.47; <0.001)
Interaction of suicidal behavior and race/ethnicity	
(1) and (3)	1.21 (1.00–1.48; 0.053)
(1) and (4)	1.13 (0.95–1.36; 0.165)
(2) and (3)	1.25 (0.93–1.69; 0.139)
(2) and (4)	1.19 (0.88–1.61; 0.259)
Age, years (35–49)	
18–25	0.60 (0.57–0.62; <0.001)
26–34	0.83 (0.80–0.87; <0.001)
50–64	0.89 (0.85–0.93; <0.001)
⩾65	0.28 (0.26–0.31; <0.001)
Sex (female)	
Male	0.51 (0.49–0.53; <0.001)
Marital status (married)	
Widowed	1.02 (0.93–1.10; 0.721)
Divorced/separated	1.42 (1.35–1.49; <0.001)
Never married	1.17 (1.12–1.23; <0.001)
Education (<high school)	
High school or equivalent	1.04 (0.98–1.11; 0.170)
Some college	1.35 (1.27–1.44; <0.001)
⩾Bachelor's degree	1.69 (1.58–1.80; <0.001)
Employment (full-time)	
Part-time	1.28 (1.22–1.34; <0.001)
Unemployed	1.35 (1.27–1.43; <0.001)
Other	1.52 (1.46–1.58; <0.001)
Family income (<$20 000)	
$20 000–$49 999	0.92 (0.88–0.97; 0.001)
$50 000–$74 999	0.92 (0.86–0.97; 0.004)
⩾$75 000	0.96 (0.91–1.01; 0.090)
Insurance coverage (private plan)	
Medicaid	1.26 (1.20–1.34; <0.001)
Medicare	2.05 (1.90–2.21; <0.001)
Other	1.41 (1.32–1.51; <0.001)
Uninsured	0.62 (0.59–0.66; <0.001)
Urban residence (non-metropolitan)	
Small metropolitan	1.14 (1.09–1.19; <0.001)
Large metropolitan	1.10 (1.05–1.15; <0.001)
Past-year major depressive episode (no)	6.53 (6.27–6.81; <0.001)
Past-year substance use disorder (no)	
Alcohol	1.53 (1.45–1.62; <0.001)
Tobacco	1.28 (1.22–1.34; <0.001)
Cannabis	1.32 (1.21–1.45; <0.001)
Cocaine	1.14 (0.94–1.38; 0.188)
Opioid	1.90 (1.66–2.18; <0.001)
Hallucinogen	1.36 (0.94–1.95; 0.098)
Stimulant	1.74 (1.38–2.19; <0.001)
Sedative	1.97 (1.56–2.49; <0.001)
Survey year (2009–2010)	
2011–2012	1.06 (1.01–1.12; 0.018)
2013–2014	1.12 (1.06–1.18; <0.001)
2015–2016	1.04 (0.99–1.09; 0.160)
2017–2018	1.09 (1.03–1.14; 0.002)
2019–2020	1.22 (1.16–1.29; <0.001)

*Note*: Data are from National Survey on Drug Use and Health. AOR, adjusted odds ratio; CI, confidence interval.

Among those who did not receive services, Black and Hispanic individuals reporting SI were more likely to report not knowing where to go for mental health services (32.6% and 40.2%, respectively) and Hispanic individuals were more likely to report fearing that others would know they had received services (Table 4). Among those who made a past-year SA, Hispanic individuals were more likely to think they could handle without treatment and to fear that others would know they had received services.

**Table 4.** Reasons for not getting mental health treatment among those who did not receive mental health care by race/ethnicity and suicidal behavior, 2009–2020

	Non-Hispanic white	Non-Hispanic black	Hispanic (white or black)	*p*
*Suicidal ideation*	***n* = 7534**	***n* = 2266**	***n* = 3230**	
Reasons for not getting mental health treatment				
Couldn't afford cost	53.0%	52.6%	46.0%	0.150
Concern about effect on job	14.7%	13.1%	12.4%	0.697
Insurance did not cover mental health care	8.4%	6.2%	8.2%	0.510
Concern about confidentiality	14.6%	13.6%	14.2%	0.934
Didn't think treatment would help	14.4%	7.3%	14.7%	0.049
Thought could handle without treatment	27.1%	18.1%	27.1%	0.061
Didn't know where to go for treatment	26.8%	32.6%	40.2%	0.001
Didn't have time	15.3%	12.8%	14.5%	0.717
Didn't want others to find out	11.1%	6.9%	17.5%	0.001
Lack of transportation or services too far away	4.4%	5.9%	5.2%	0.645
Concern about opinions of others	16.0%	12.4%	16.8%	0.543
*Suicide attempt*	***n* = 820**	***n* = 349**	***n* = 411**	
Reasons for not getting mental health treatment				
Couldn't afford cost	49.7%	53.4%	40.5%	0.494
Concern about effect on job	14.3%	13.5%	7.1%	0.434
Insurance did not cover mental health care	7.1%	4.8%	7.0%	0.796
Concern about confidentiality	15.5%	7.0%	10.4%	0.143
Didn't think treatment would help	12.0%	7.2%	15.4%	0.399
Thought could handle without treatment	17.4%	11.6%	36.0%	0.004
Didn't know where to go for treatment	31.9%	39.7%	34.9%	0.693
Didn't have time	12.6%	5.6%	20.3%	0.081
Didn't want others to find out	7.7%	1.0%	16.9%	0.004
Lack of transportation or services too far away	7.7%	10.1%	6.2%	0.827
Concern about opinions of others	16.3%	12.8%	14.4%	0.807

*Note*: Data are from National Survey on Drug Use and Health.

Bold indicates sample sizes by suicidal behavior.

## Discussion

In this nationally representative sample of US adults from 2009 to 2020, Black and Hispanic individuals were significantly *less* likely than White individuals to report past-year SI but among individuals with past-year SI, were significantly *more* likely to report a past-year SA, a result that was essentially unchanged after controlling for a large number of potentially confounding sociodemographic and clinical factors. Stated differently, Black and Hispanic individuals report less frequent *thought*s of suicide than White individuals but when they do, they are more likely to report *active attempts*. Stark differences were also found in use of mental health services with *less* use by Black and Hispanic individuals. However, interaction analyses suggested no significant relationship between service use and racial/ethnic differences in SI or SA. There were few significant differences between racial/ethnic groups who did not use mental health services in the *reasons* for not using such services. Black and Hispanic individuals were more likely to report not knowing where to go for treatment and Hispanic individuals were more likely to report concerns about stigma.

While much attention has been focused on the recent increase in SAs and suicide deaths among Black adolescents (Congressional Black Caucus, 2019; Xiao, Cerel, & Mann, 2021), very little attention has been paid to racial/ethnic differences in suicidal behavior among adults, despite the observed increase in suicide rates among Black and Hispanic adults in data from the National Center for Health Statistics (Curtin, 2022; Garnett et al., 2022). Our results provide updated epidemiologic estimates of racial and ethnic differences in past-year SI and SAs and are believed to be the first study in adults to show that among individuals with past-year SI, Black adults may be more likely than Whites to report a past-year SA. These findings are consistent with several recent studies of adolescents showing that Black and Hispanic adolescents are less likely to report past-year SI but may be more likely to report SAs (Lindsey, Sheftall, Xiao, & Joe, 2019; Romanelli, Xiao, & Lindsey, 2020; Xiao, Romanelli, & Lindsey, 2019). Data from older epidemiologic surveys of adults, including the ECA, NCS, and NCS-R, either found no significant racial/ethnic difference or greater rates of SAs among White adults (Baca-Garcia et al., 2010; Kessler et al., 2005; Moscicki et al., 1988; Olfson et al., 2017). In addition, data from the NSAL and NLAAS examined lifetime SI and SAs but did not directly compare suicidal thoughts/behavior across racial/ethnic groups (Cheng et al., 2010; Joe et al., 2006, 2009). It is important to note that our findings on SAs do not generalize to the general population as past-year SAs were only assessed among individuals who reported past-year SI, not among the entire survey sample. Another potential explanation for our diverging results include the possibility that SAs among Black individuals may have disproportionately increased in recent years compared to other racial groups, although we did not find evidence supporting this possibility. This explanation may find support from the finding of a recent study that past-year SAs among Black adults with MDE have nearly doubled and increased more than any other demographic subgroup (Bommersbach, Rosenheck, & Rhee, 2022b) [under review]. It is also possible that, even in the absence of a differential temporal trend, our use of a larger sample size resulted in greater power to detect differences between racial/ethnic groups.

Our results demonstrating that when Black and Hispanic individuals report suicidality, they are much more likely to report more severe suicidal behavior and less likely to report SI, and it is alarming given the recent rise in suicide deaths among Black and Hispanic individuals (Curtin, 2022). An often-cited explanation for this rise in suicide is that minoritized populations may be less likely to receive mental health care when experiencing suicidality, either due to reduced help-seeking behavior (Taylor & Kuo, 2019), concerns about stigma (Nadeem et al., 2007), greater comfort with alternative treatment options, or structural barriers (Lillie-Blanton & Hoffman, 2005). While our results demonstrate that Black and Hispanic individuals are less likely to receive mental health services, this did not account for the greater frequency of SA. Thus, other explanations are likely contributing to these findings.

Reporting styles may partially explain these results. Black and Hispanic individuals may be less likely to report suicidal thoughts or behavior until they reach a more severe threshold, either due to differences in socio-cultural norms for the expression of distress, perhaps reflecting greater mental health stigma. Our study clearly found Hispanic individuals experiencing suicidality were particularly concerned about stigma. Second, it is possible that disparities in aspects of service use not measured in this study may contribute to the findings. For example, Black and Hispanic individuals may be less likely to receive evidenced-based treatment or timely treatment in proximity to need, either due to possible provider bias and structural racism or less frequent expression of suicidality, as our study may suggest (Wang et al., 2005). Prior studies have demonstrated that Black patients are more likely to experience mental health treatment delays, less likely to receive prescription medication for depression, and report less satisfaction with services (Alegria et al., 2008; Bommersbach, Jegede, Stefanovics, Rhee, & Rosenheck, 2022a; Cook et al., 2014, 2019; Maura & Weisman de Mamani, 2017; McFarland & Klein, 2005; Olfson, Cherry, & Lewis-Fernandez, 2009; Remmert, Guzman, Mavandadi, & Oslin, 2022; Wang et al., 2005; Wells, Klap, Koike, & Sherbourne, 2001). Additionally, our results suggest that Black and Hispanic individuals with SI are less likely to know where to go for treatment, suggesting a failure of the mental health system to ensure services are known and approachable for these populations. A better understanding of how these well-documented disparities impact outcomes among minoritized populations with suicidal thoughts or behavior are needed (Washington, 2006).

These results have a number of potential implications for suicide prevention. First, providers should be aware of increasing rates of suicide and higher rates of SAs among Black and Hispanic individuals. Training on suicide risk assessment should avoid propagating generalized beliefs that White males are most impacted by suicidality. Such teaching may result in under-recognition of suicidality among Black and Hispanic individuals. In addition, the results of our study raise the question of whether Black and Hispanic individuals are more likely to under-report SI. Such a finding may have impeded accurate clinical assessment of suicidality, but requires replication in clinical samples.

Additionally, as research continues to shed light on potential racial/ethnic differences in suicidal behavior, suicide prevention programs would do well to recognize differential risks for suicide across race/ethnicity, including the interpersonal and structural impacts of racism, adverse childhood experiences, and socioeconomic disparities (Washington, 2006; Xiao et al., 2021). Continued efforts to dismantle structural racism in healthcare settings is critical to ensuring that Black and Hispanic individuals experiencing suicidality feel comfortable seeking professional care and receive timely, evidence-based care. In addition, interventions outside the healthcare system to improve identification of high-risk individuals are needed, including training in suicide risk assessment for clergy, religious leaders, and school personnel; public health promotion campaigns that target mental health stigma, especially among Hispanic populations; and continued expansion of community-based crisis services.

Several potential limitations deserve mention. Most notably, the retrospective, self-report nature of the data introduces the possibility of recall and reporting bias. Future research could consider examining medically-verifiable SAs to mitigate this limitation. In addition, SAs were only assessed among individuals reporting past-year SI so, as stated previously, our findings on SAs are only applicable to individuals with SI. In addition, SAs were assessed with a single question that may have mis-classified non-suicidal self-injury. Third, NSDUH does not survey individuals who are currently incarcerated. Given that racial minorities are over-represented in prisons and jails, rates of SI and SAs may have been underestimated in this study. Finally, the broad racial and ethnic categories available in NSDUH did not allow for more nuanced understanding of racial and ethnic subgroups (Jegede, Na, Rhee, Stefanovics, & Rosenheck, 2021).

In spite of these limitations, this nationally representative study suggests that Black and Hispanic individuals are less likely than White individuals to report SI but when they do, they are more likely to report attempts. While they are less likely to report receiving mental health treatment, less service use does not underlie the difference in suicidal behavior across racial/ethnic groups. Thus, it is likely that other factors, including differences in reporting styles, socio-culturally acceptable forms of distress, and possible structural racism in the mental health system, may differentially impact racial/ethnic subgroups and risk of both suicide precursors and suicide mortality. Further study of these phenomena is needed.

## Data

The data have not been previously presented orally or by poster at scientific meetings.
